# Molecularly Imprinted Polymer-Based Electrochemical BioSensors for *Haemophilus influenzae* Rapid Detection

**DOI:** 10.3390/polym18060726

**Published:** 2026-03-17

**Authors:** Naphatsawan Vongmanee, Jindapa Nampeng, Chuchart Pintavirooj, Sarinporn Visitsattapongse

**Affiliations:** Department of Biomedical Engineering, School of Engineering, King Mongkut’s Institute of Technology Ladkrabang, Bangkok 10520, Thailand; naphatsawan.v@hotmail.com (N.V.); jindapa.na@kmitl.ac.th (J.N.); chuchart.pi@kmitl.ac.th (C.P.)

**Keywords:** *Haemophilus influenzae*, molecular imprinted polymers technique, electrochemical sensors

## Abstract

*Haemophilus influenzae* (*H. influenzae*) is an important respiratory pathogen that can cause various invasive and non-invasive bacterial infections requiring rapid and sensitive detection. In recent years, electrochemical biosensors have emerged as a practical alternative for pathogen detection due to their high sensitivity, portability and short analysis time. Molecularly imprinted polymers (MIPs) are a class of synthetic receptors designed to mimic biological recognition through template-directed polymerization. In this study, an electrochemical biosensor based on MIPs was developed for the selective detection of *H. influenzae.* The polymeric film composed of methacrylamide (MAM), acrylamide (AAM), and vinylpyrrolidone (VP) monomers was fabricated on a gold screen-printed electrode (gold-SPE). The results of cyclic voltammetry (CV) revealed a strong redox current shift corresponding to bacteria concentrations within an analytical range of 1–10,000 CFU/mL with LOD 1.03 CFU/mL, with relative standard deviation (RSD) values below 9% across the tested concentration range. The optimized composition yielded and exhibited excellent selectivity when tested against non-target bacteria such as *Klebsiella pneumoniae*, *Pseudomonas aeruginosa*, and *Staphylococcus aureus*.

## 1. Introduction

*H. influenzae* is a small Gram-negative coccobacillus approximately 1.0 × 0.3 µm in size that colonizes the human upper respiratory tract and is a major causative agent of respiratory infections such as otitis media, sinusitis, bronchitis, and pneumonia [1]. *H. influenzae* is also associated with severe diseases including meningitis and septicemia [2], especially in infants and immunocompromised individuals [3]. Recent epidemiological studies estimate that *H. influenzae* infections account for over 3 million serious illnesses and approximately 400,000 deaths annually [4].

Conventional diagnostic methods for *H. influenzae* include bacterial culture, polymerase chain reaction (PCR) and enzyme-linked immunosorbent assay (ELISA), which provide reliable results [5]. Although these techniques are sensitive and specific, they require sophisticated laboratory infrastructure, trained personnel, and are time-consuming and labor-intensive. Moreover, bacterial culture can take time up to 48 h to produce results, which may delay clinical decision-making regarding appropriate antibiotic treatment and infection control for *H. influenzae* [6]. These limitations highlight the critical need for rapid detection that is cost-effective and suitable for point-of-care applications [7].

Currently, biosensor technology has emerged as a promising platform for bacterial pathogen detection and has been widely applied in biomedical diagnosis as well as a wide range of other areas due to their high sensitivity, rapid response time, and portability [8,9,10]. Among various biosensing approaches, molecularly imprinted polymers (MIPs) offer several advantages over biological recognition elements such as antibodies and enzymes, including greater chemical stability, lower cost, and reusability [11,12,13,14,15,16]. MIPs are synthetic polymers with specific recognition sites formed by polymerizing monomers in the presence of a target template [17,18], which is later removed to create complementary binding cavities [19,20]. MIP techniques involve synthetic materials engineered to function as selective receptors for specific target molecules [21,22]. This is achieved by allowing functional monomers to self-organize around a bacterial template followed by polymerization in the presence of a crosslinking agent [23,24]. After the polymerization process, the template is extracted, leaving behind highly specific cavities within the polymer matrix [25,26]. These cavities are structurally and chemically complementary to the target bacteria, enabling selective binding of the original template or closely related species [27,28].

This study reports the fabrication and characterization of MIP-based electrochemical biosensor for the selective detection of *H. influenzae*. The MIP layer was electropolymerized onto a gold screen-printed electrode using methacrylamide (MAM), acrylamide (AAM), and vinylpyrrolidone (VP) as functional monomers to construct selective recognition sites. The electrochemical performance of the fabricated sensor was evaluated using cyclic voltammetry (CV), demonstrating high sensitivity and specificity towards *H. influenzae* with minimal interference from non-target bacteria. This platform represents a promising step towards rapid, portable and cost-effective detection of *H. influenzae*, addressing the need for effective diagnostic tools in resource-limited settings. The sensor demonstrated a dynamic detection range from 1 to 10,000 CFU/mL with a limit of detection (LOD) as low as 1.0294 CFU/mL.

## 2. Materials and Methods

### 2.1. H. influenzae Preparation and Fixation Procedure

*H. influenzae* was cultured in Luria Bertani (LB) broth (HiMedia Laboratories, Mumbai, India) by isolating a single colony from the stock culture and transferring it into 5 mL of LB broth. The culture was incubated at 37 °C for 16 h to promote optimal bacterial growth [29]. Subsequently, tenfold serial dilutions were carried out, followed by colony enumeration using the spread plate method. To preserve and maintain the bacterial cells, they were fixed using 2.5% glutaraldehyde (25%, Sigma-Aldrich, St. Louis, MO, USA) for 2.5 h at room temperature to stabilize the cellular structures. After fixation, the bacteria cells were rinsed with phosphate-buffered saline (PBS) (Merck, Darmstadt, Germany) to remove excess fixative. Subsequently dehydration was performed through a graded ethanol series (99.99%, Merck, Darmstadt, Germany), involving sequential exposure to ethanol concentrations of 30%, 50%, 70%, 80%, 90%, and 95%, each for 15 min. This was followed by two washes in 100% ethanol, each for 15 min [30]. Finally, the bacteria cells were resuspended in PBS to obtain the final suspension. This fixation method ensured the structural integrity of the cells for effective MIP template imprinting.

### 2.2. The Number of Imprinting H. influenzae on Electrode

**Figure 3:**
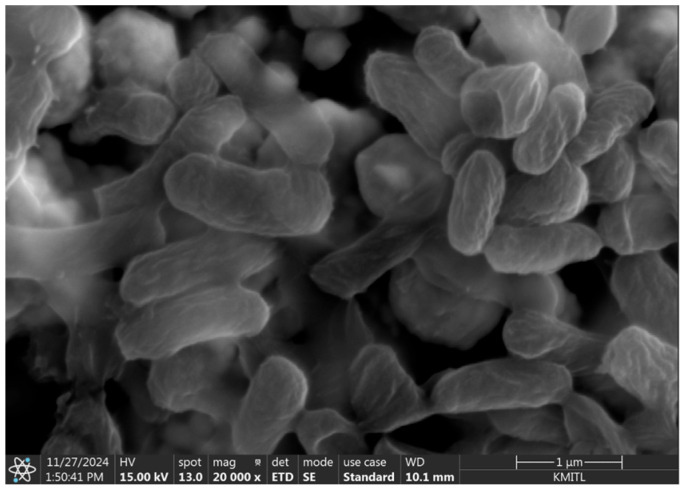
Scanning electron microscope (SEM) image showing the morphology of *H. influenzae* cells observed on the electrode surface at a magnification of 20,000×.

Prior to imprinting, the theoretical number of *H. influenzae* cells that could occupy the working electrode surface was estimated based on the ratio between the working electrode area and the average size area of a single bacterial cell. The working electrode area was calculated geometrically (πr^2^) with a diameter of 4 mm (DRP-220BT, Dropsens, Asturias, Spain), whereas the average bacterial cell area was approximated from SEM images, as shown in Figure 3. The average cell size was approximately 0.3 × 1.00 µm. So, the average size of bacteria area is 0.236 µm^2^ per cell, calculated based on an elliptical approximation of the cell shape. From this calculation, the total number of bacteria that could theoretically be imprinted on the electrode surface was estimated to be 5.32 × 10^7^ cells. However, since bacterial cells cannot perfectly arrange to fully cover the electrode surface due to steric hindrance and irregular orientation, this value represents only the maximum theoretical density. To account for this limitation, the applied bacterial suspension was diluted to approximately tenfold of the theoretical concentration corresponding to 5.32 × 10^6^ cells to allow effective imprinting while preventing overcrowding of cells on the electrode surface.

### 2.3. Polymer Synthesis for H. influenzae Detection

A molecularly imprinted polymer (MIP) layer was fabricated on the surface of the working electrode to achieve selective recognition toward *H. influenzae*. The functional monomers used in this work included methacrylamide (MAM), acrylamide (AAM), and vinylpyrrolidone (VP), which were selected due to their complementary chemical functionalities and strong affinity for bacterial surface components. The monomers were co-polymerized in the presence of the *H. influenzae* template to generate recognition sites with high specificity. Polymerization was carried out under five different synthesis conditions to optimize the performance of the MIPs layer and to investigate the effect of functional monomer composition on imprinting efficiency and electrochemical performance. The selected ratios represent varying relative proportions of MAM, AAM, and VP to evaluate the influence of hydrogen-bonding capacity and hydrophilicity on bacterial recognition, as summarized in Table 1.

For the preparation of the pre-polymer gel solution, N,N′-(1,2-dihydroxyethylene)bisacrylamide (DHEBA) was employed as the cross-linker, while dimethyl sulfoxide (DMSO) served as the solvent [31]. The functional monomers (MAM, AAM, and NVP) were mixed with 45 mg DHEBA and 300 µL DMSO. The mixture was subsequently heated at 70 °C for 15 min to facilitate the formation of a pre-polymer gel solution [32], which was then subjected to polymerization in the presence of the *H. influenzae* template.

### 2.4. Screen-Printed Electrode Preparation (SPE)

**Figure 1:**
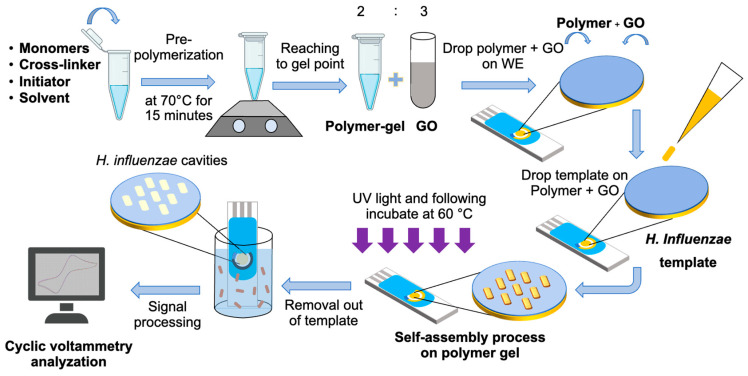
Schematic illustration of the fabrication process of the molecularly imprinted polymer (MIP)-based electrochemical biosensor for *H. influenzae* detection. The process involves pre-polymerization of monomers with cross-linker, initiator, and solvent, mixing with GO, deposition onto the working electrode, imprinting with *H. influenzae* templates, UV/thermal polymerization, template removal, and subsequent electrochemical analysis by cyclic voltammetry.

The SPE employed in this study (DRP-220BT, Dropsens, Asturias, Spain) comprises three electrode configurations, including a gold working electrode, a gold counter electrode, and a silver reference electrode. The pre-polymer gel obtained in Section 2.3 was combined with graphene oxide (GO) at a concentration of 0.15 mg/mL to enhance the electrical conductivity of the polymer matrix at a ratio of 2:3 [33]. The resulting composite was deposited onto the working electrode surface to form a thin sensing layer. Subsequently, *H. influenzae* templates were introduced onto the pre-polymer/GO layer and allowed to interact with the matrix. Afterward, the screen-printed electrode was exposed to UV irradiation for 3 h to initiate crosslinking and imprinting processes, resulting in the formation of a stable molecularly imprinted-on sensing film [34]. This was followed by incubation at 60 °C for 18 h to complete the polymerization [35]. The overall fabrication process is illustrated schematically in Figure 1.

**Figure 4:**
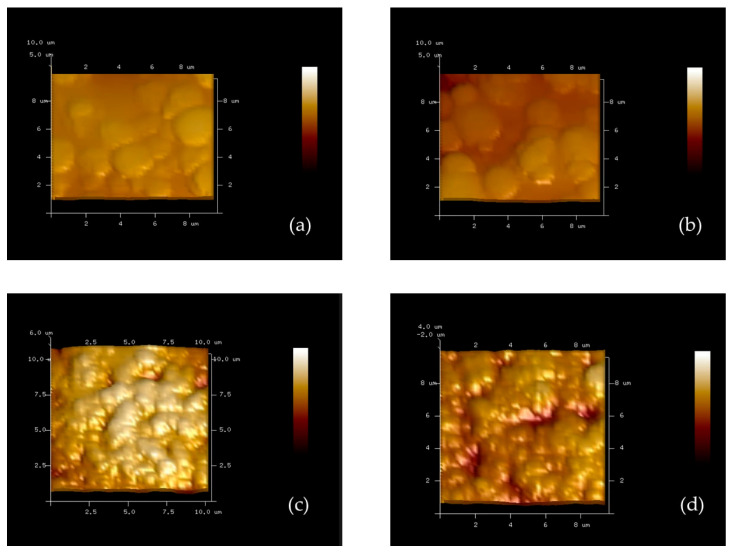
Atomic force microscopy (AFM) images illustrating surface morphology at different stages of electrode modification: (**a**) bare gold screen-printed electrode (SPE) surface, (**b**) polymer-coated SPE surface, (**c**) *H. influenzae* imprinting on the polymer surface, and (**d**) formation of *H. influenzae* cavities after template removal.

After fabrication, the SPE was washed out of the *H. influenzae* template by soaking in a 10% acetic acid solution for 30 min and followed by soaking with DI water for an additional 30 min to ensure complete template removal. To confirm the successful removal of *H. influenzae* template and the integrity of the imprinted cavities, surface characterization was conducted using a scanning electron microscope. The images showed clearly defined cavities corresponding to the size and morphology of *H. influenzae*. In addition, comparison between *H. influenzae* imprinted on electrode and bare electrode demonstrated distinct surface morphology, confirming the preservation of the imprint structure as shown in Figure 4.

## 3. Results

### 3.1. Initial H. influenzae Stock Concentration

**Figure 2:**
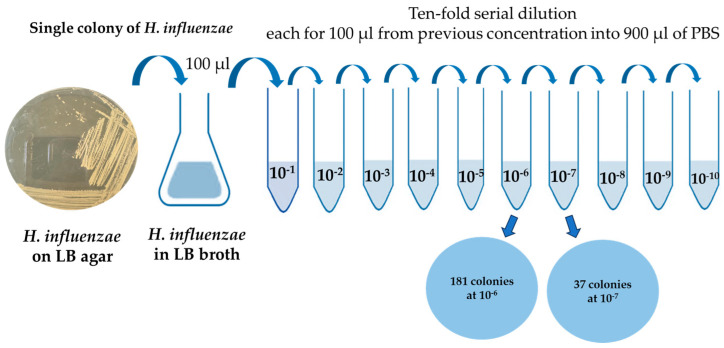
The schematic of tenfold serial dilution protocol.

From the serial dilution and spread plate technique mentioned in Section 2.1, the procedure is shown in Figure 2. The dilution at 10^−7^ yielded 37 colonies. Based on the colony counting principle, the initial bacterial stock concentration (CFU/mL) can be calculated using the formula:

(1) CFUmL= Number of colonies×Dilution factorVolume plated (mL)

In this experiment, the plated volume was 0.1 mL. Therefore, the initial *H. influenzae* stock concentration was estimated to be approximately 3.7 × 10^9^ CFU/mL. This concentration was used as the reference stock for subsequent imprinting experiments.

### 3.2. Morphological Characterization of H. influenzae by SEM

The morphology of *H. influenzae* was examined using scanning electron microscopy (SEM) as shown in Figure 3. The SEM image reveals small rod-shaped bacterial cells with smooth surfaces, which are consistent with the typical morphological characteristics of *H. influenzae*. The observed cell size and shape confirm the structural integrity of the bacterial cells used in this study. The average bacterial cell size was approximately 0.3 × 1.00 µm.

### 3.3. Surface Characterization of Electrodes

The surface morphology of the electrodes at different modification stages was examined using atomic force microscopy (AFM) to evaluate the polymer coating, bacterial imprinting, and cavity formation processes.

As shown in Figure 4, the bare gold SPE (a) shows a relatively smooth and uniform surface with small granular structures. After polymer modification (b), the surface became more homogeneous, indicating the successful formation of a uniform polymer film on the electrode surface. Following exposure to *H. influenzae* (c), the polymer surface exhibited increased roughness and irregular structures corresponding to the shape and size of bacterial cells, suggesting successful bacterial imprinting within the polymer matrix. After the removal of the bacterial template (d), distinct cavities resembling the morphology of *H. influenzae* were clearly visible. The observed cavities exhibited an average lateral dimension of approximately 0.7 × 0.2 µm and a depth of around 2 µm. The lateral dimensions fall within the reported size range of *H. influenzae* (0.5–1.0 µm), supporting successful imprint formation. The slightly greater cavity depth compared to the reported bacterial size may result from gold substrate roughness on SPE and AFM measurement artifacts rather than the actual bacterial dimension. These cavities confirm the successful imprinting process and demonstrate that the polymer surface retained specific recognition sites capable of selectively rebinding the target bacteria. Overall, the AFM results verify the effective fabrication of the molecularly imprinted polymer (MIPs) layer and its potential for selective bacterial detection.

### 3.4. CV Characterization of H. influenzae Interaction

**Figure 5:**
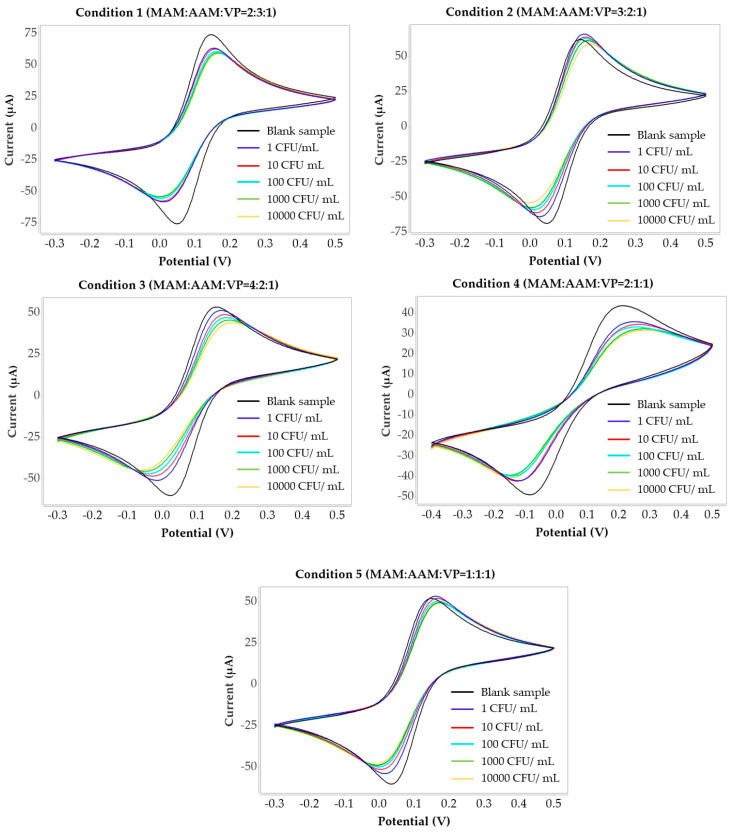
Cyclic voltammograms recorded for *H. influenzae* at concentration 1–10^4^ CFU/mL using MIP sensors synthesized under five polymer conditions.

The electrochemical behavior of *H. influenzae* samples was investigated using cyclic voltammetry (CV) with a screen-printed electrode in the presence of a ferri/ferrocyanide redox couple. The redox peaks observed in the CV curves correspond to the reversible redox reaction of the electrochemical probe, potassium ferri/ferrocyanide, present in the supporting electrolyte solution. The well-defined anodic and cathodic peaks are attributed to the electron transfer process of the redox couple at the electrode surface. The MIP layer plays a modulatory role in the electron transfer process rather than acting as an electroactive species. After polymerization, the presence of the MIP film partially hinders the diffusion of the redox probe toward the electrode surface, resulting in a decrease in peak current compared to the bare electrode. Upon binding of *H. influenzae* to the imprinted cavities, the captured bacterial cells further obstruct the mass transport of the ions and increase the interfacial electron transfer resistance. This leads to a noticeable reduction in peak current and changes in peak-to-peak separation. Figure 5 shows representative CV curves for five polymer synthesis conditions, measured at *H. influenzae* concentrations ranging from 1 to 10,000 CFU/mL. The anodic peak current increased with increasing bacterial concentration, indicating a clear correlation between the presence of *H. influenzae* and the electrochemical response. Blank measurements (electrolyte without bacteria) exhibited stable CV curves with the highest peak currents, confirming that the absence of bacteria leads to minimal resistance at the electrode surface and consequently maximal current response. In contrast, increasing concentrations of *H. influenzae* promoted bacterial adhesion to the electrode surface, which altered the interfacial properties and increased the electron-transfer resistance. This effect led to a gradual decrease in peak current compared with the blank. The observed electrochemical signals can therefore be attributed to the specific interactions between the bacterial cells and the electrode surface.

The current responses at different bacterial concentrations were compared to those of the blank sample, and the results were expressed as the percentage of relative current change to reflect variations at each concentration level. To further assess and compare electrode performance, the percentage change in current was calculated as the relative difference between the response obtained in the presence of *H. influenzae* and that of the blank. This provided a normalized parameter for evaluating sensitivity across the tested concentration range.

(2) $$Relative\;Current\;Changing\;\left(\%\right)=\frac{\left(I-I_{0}\right)}{I_{0}}\times 100$$

where I is the current for concentration levels of *H. influenzae* and I_0_ is the current of the blank electrode.

**Figure 6:**
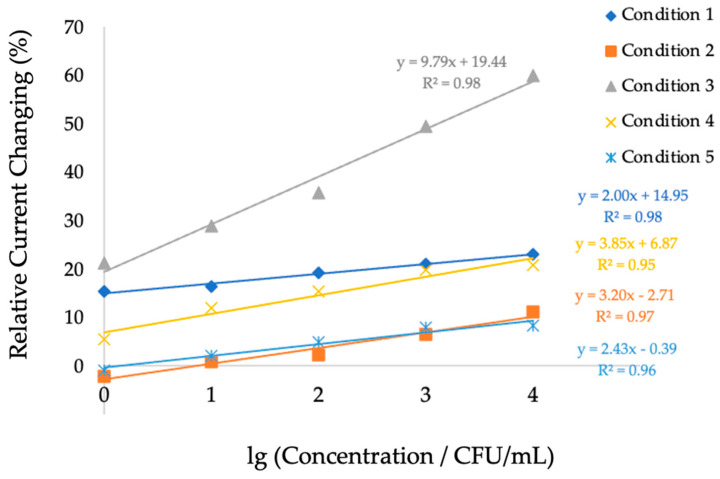
Comparison of relative current changing (%) of *H. influenzae* at different concentrations (CFU/mL) in five polymer synthesis conditions.

Comparisons among the five polymer synthesis conditions were performed, and the linear regression analysis is shown in Figure 6.

Figure 6 presents the relationship between the logarithm of *H. influenzae* concentration (CFU/mL, x-axis) and the relative current changing (µA, y-axis). All five polymer conditions exhibited positive electrochemical responses with increasing *H. influenzae* concentrations. The results from condition 3 consisting of MAM:AAM:VP in a ratio of 4:2:1 provided the best performance with the highest sensitivity. This is evidenced by its slope (9.79) and strong correlation coefficient (R^2^ = 0.98) compared to the other conditions that exhibited lower responses in *H. influenzae* detection. For polymer condition 1, 2, 4 and 5 with MAM:AAM:VP at ratios of 2:3:1, 3:2:1, 2:1:1 and 1:1:1, respectively, the regression coefficients (R^2^) were 0.98, 0.97, 0.95 and 0.96, respectively.

**Figure 7:**
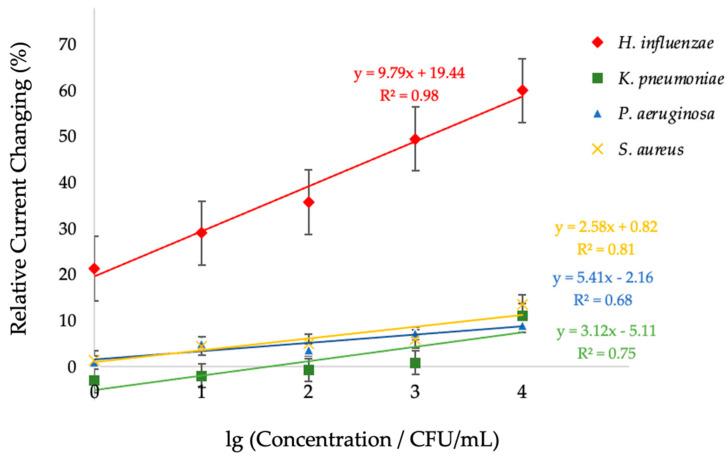
Linear relationship between percent current changing and the logarithmic concentration of *H. influenzae* under condition 3 (MAM:AAM:VP = 4:2:1) compared with the responses obtained from negative control bacteria (*K. pneumoniae*, *P. aeruginosa* and *S. aureus*).

Therefore, condition 3 was selected for the selectivity evaluation of the sensor as it demonstrated the highest sensitivity and reproducibility in detecting *H. influenzae*. To evaluate the selectivity of the developed sensor, measurements were performed using non-target bacterial strains, including *Klebsiella pneumoniae* (*K. pneumoniae*), *Pseudomonas aeruginosa* (*P. aeruginosa*), and *Staphylococcus aureus* (*S.aureus*), under the same concentration range as *H. influenzae*. The current responses obtained from these negative control strains were significantly lower than those from *H. influenzae*, demonstrating the sensor’s high specificity toward the target bacterium. The linear regression analysis is shown in Figure 7.

The non-target bacteria exhibited only slight variations in current response compared to the pronounced signals generated for *H. influenzae* within the tested concentration range of 1–10,000 CFU/mL. Among the examined species, the biosensor displayed the greatest sensitivity toward *H. influenzae*, achieving 9.79% relative current changing per logarithmic increase in concentration. In contrast, the sensitivities determined for *K. pneumoniae*, *P. aeruginosa*, and *S. aureus* were 3.12, 5.41, and 2.58% relative current changing per logarithmic increase in concentration, respectively. The response intensity for *H. influenzae* exhibited a clear linear correlation with concentration, whereas the signals from the non-target bacteria remained minimal and displayed no significant linear trend. This result confirms that the polymer modified in condition 3 on screen-printed electrode effectively discriminates *H. influenzae* from other bacterial species, which is essential for reliable pathogen detection in biological samples.

### 3.5. Repeatability Evaluation

**Figure 8:**
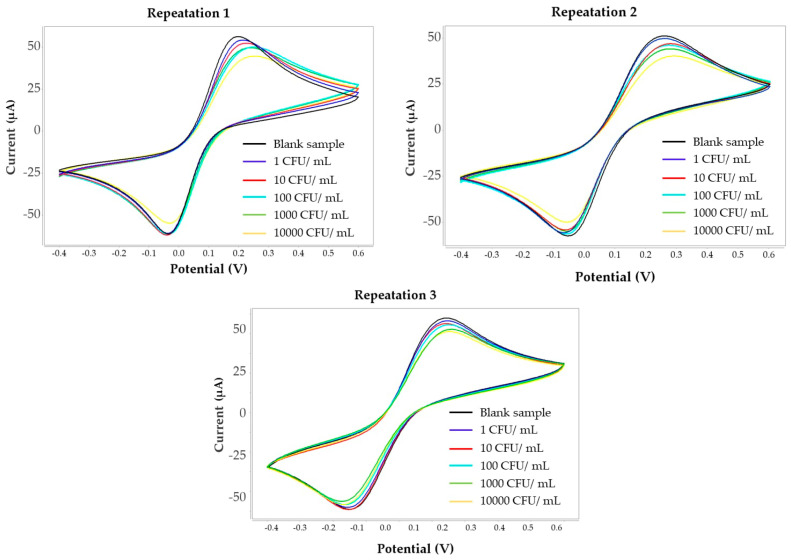
Cyclic voltammograms showing the repeatability of the MIP-based electrochemical sensor at different concentrations of *H. influenzae* 1–10,000 CFU/mL, measured in triplicate under polymer synthesis condition 3.

The repeatability was assessed by performing three measurements during concentration 1–10,000 CFU/mL of *H. influenzae* by the same sensor under polymer synthesis in condition 3. The relative current changing of the peak current response values for all concentrations are shown in Table 2. The corresponding cyclic voltammetry curves are presented in Figure 8.

The relative standard deviation (RSD) of the peak current response was calculated to range from 2.10% to 9.05% across the concentration range of 1–10,000 CFU/mL, as shown in Table 3, indicating acceptable measurement stability and reliable electrochemical performance of the developed MIP-based biosensor.

### 3.6. Limit of Detection (LOD)

The limit of detection (LOD) of the proposed MIP-based electrochemical biosensor was calculated based on the standard deviation of the blank signal and the slope of the calibration curve, according to the equation:

LOD = 3σ/S(3)

where σ represents the standard deviation of the blank sample response and S is the slope of the linear calibration plot. Based on this calculation, the LOD was determined to be 1.03 CFU/mL, indicating the high sensitivity of the developed sensor for the detection of *H. influenzae*.

### 3.7. Comparison with Reported Method

To further assess the analytical performance of the developed MIP-based electrochemical biosensor, the LOD and linear range were compared with previously reported study methods for *H. influenzae* detection. The comparison is summarized in Table 4.

The proposed sensor exhibited a wide linear detection range of 1–10,000 CFU/mL and a low limit of detection (LOD) of 1.03 CFU/mL. Compared to recently reported electrochemical aptasensor for bacterial detection, such as the platform developed by [39] targeting foodborne pathogens with an LOD of approximately 50 CFU/mL, the present MIP-based sensor demonstrates superior sensitivity.

Similarly, a recent MIP-based electrochemical sensor for *E. coli* reported by [40] achieved an LOD of approximately 9.40 CFU/mL, which remains higher than that obtained in this work. Electrochemical immunosensors reported in 2024 for pathogenic bacteria detection have shown detection limits in the range of 8 CFU/mL. However, these systems rely on biological recognition elements such as antibodies, which may suffer from limited stability and higher production costs.

Although the compared platforms target different bacterial species, they represent recent state-of-the-art electrochemical sensing strategies and provide relevant performance benchmarks. Overall, the developed MIP-based electrochemical biosensor demonstrates competitive analytical performance, combining high sensitivity, a broad dynamic range, operational simplicity, and the absence of biological recognition components, highlighting its potential for rapid and cost-effective detection of *H. influenzae*.

## 4. Discussion

The electrochemical detection of *H. influenzae* using polymer-modified screen-printed electrode demonstrated that the polymer composition plays a critical role in determining the overall sensitivity of the biosensor. Among the five tested polymer conditions, the electrode fabricated under condition 3 (MAM:AAM = 4:2:1) exhibited the highest current response and strongest linear correlation with bacterial concentration, with a slope of 9.79 and a correlation coefficient (R^2^) of 0.98.

The superior performance of this condition toward *H. influenzae* detection can be attributed to the synergistic interaction among the three monomer components. MAM provides amide groups (–CONH_2_) capable of forming strong hydrogen bonds with the bacterial cell wall components, particularly with peptidoglycan and lipooligosaccharides found in *H. influenzae*. AAM, which also contains amide functional groups, contributes additional hydrogen bonding sites and enhances the overall hydrophilicity of the polymer surface, facilitating efficient bacterial adhesion and charge transfer [41].

Both MAM and AAM contain amide groups that facilitate hydrogen bonding and electrostatic interactions with bacterial cell wall components, promoting bacterial adsorption onto the electrode surface. The slightly higher MAM content in condition 3 likely provides a more stable and less swollen polymer network, preventing excessive hydration that could hinder electron transfer. Meanwhile, VP contributes to film formation and enhances surface wettability and ionic conductivity, allowing better contact between the redox mediator and the electrode surface [42]. The optimized 4:2:1 ratio of MAM:AAM:VP provides an optimal balance between mechanical stability, hydrophilicity, and electroactive surface sites, enabling efficient interaction between the bacterial surface and screen-printed electrode.

Moreover, the higher MAM content promotes a denser distribution of amide functionalities, enhancing the specific binding affinity toward *H. influenzae* through electrostatic and hydrogen-bond interactions. This results in more pronounced changes in current response upon bacterial binding, leading to superior sensitivity compared with other polymer formulations. AFM images of the electrode surface before and after bacterial imprinting clearly demonstrate significant morphological changes. After the imprinting of *H. influenzae*, distinct cavity-like structures corresponding to the *H. influenzae* dimensions (approximately 0.7 × 0.2 µm) were observed. These imprinted cavities reflect the successful formation of bacterial templates within the polymer matrix.

Although the sensor demonstrated excellent analytical performance under controlled laboratory conditions, further validation using real clinical samples is necessary to fully establish its practical applicability. Matrix effects and potential interferents in complex biological samples will be systematically investigated in future studies.

## 5. Conclusions

In this study, a polymer-based electrochemical biosensor composed of MAM, AAM, and VP monomers was successfully developed for the detection of *H. influenzae*. Among the five polymer compositions investigated, the formulation with a monomer ratio of MAM:AAM = 4:2:1 (Condition 3) exhibited the best electrochemical performance, providing the highest current response and sensitivity, with a strong linear correlation across a wide range of bacterial concentrations. The superior sensing behavior of the 4:2:1 formulation can be attributed to the synergistic interaction among MAM, AAM, and VP. This optimized ratio enhances hydrogen bonding capability, hydrophilicity, and surface charge distribution, thereby promoting stronger bacterial adhesion and efficient electron transfer at the electrode interface. The selectivity of the optimized sensor was also evaluated using other bacterial species (*K. pneumoniae*, *P. aeruginosa* and *S. aureus*) as negative control bacteria. The current responses from these non-target organisms were significantly lower than those observed for *H. influenzae*, confirming the high specificity of the developed sensor. Overall, the MAM:AAM (4:2:1) polymer-based biosensor demonstrated excellent sensitivity, selectivity, and reproducibility for *H. influenzae* detection. The AFM characterization confirms the successful fabrication of *H. influenzae* imprinted polymer surfaces. These findings suggest its potential as a versatile sensing platform for rapid, reliable, and cost-effective detection of bacterial pathogens through further material optimization and sensor integration.

## Figures and Tables

**Table 1:** Various conditions of polymer synthesis for *H. influenzae* detection.

Condition	Ratio (n:n)	MAM (mg)	AAM (mg)	VP (µL)
1	2:3:1	17.0	21.8	10.7
2	3:2:1	25.5	14.2	10.7
3	4:2:1	34.0	14.2	10.7
4	2:1:1	17.0	7.1	10.7
5	1:1:1	8.5	7.1	10.7

**Table 2:** Repeatability of the MIP-based electrochemical biosensor at different concentrations of *H. influenzae* (n = 3).

Repetition 1	Current (µA)	∆I (µA)	Relative Current Changing (%)
Blank	50.47		
1 CFU/mL	44.35	11.09	20.00
10 CFU/mL	39.49	15.95	28.77
100 CFU/mL	33.24	22.20	40.04
1000 CFU/mL	27.87	27.57	49.73
10,000 CFU/mL	22.23	33.21	59.91
Repetition 2	Current (µA)	∆I (µA)	Relative Current Changing (%)
Blank	48.56		
1 CFU/mL	42.45	12.99	23.43
10 CFU/mL	36.98	18.46	33.30
100 CFU/mL	34.12	21.32	38.45
1000 CFU/mL	27.56	27.88	50.29
10,000 CFU/mL	22.78	32.66	58.91
Repetition 3	Current (µA)	∆I (µA)	Relative Current Changing (%)
Blank	49.22		
1 CFU/mL	42.37	13.07	23.57
10 CFU/mL	36.52	18.92	34.13
100 CFU/mL	34.65	20.79	37.50
1000 CFU/mL	25.43	30.01	54.13
10,000 CFU/mL	21.39	34.05	61.42

**Table 3:** Average peak current and relative standard deviation (RSD) of the MIP-based electrochemical biosensor at different concentrations of *H. influenzae* (n = 3).

Concentration	Average (µA)	SD	% RSD
1 CFU/mL	22.34	2.02	9.05
10 CFU/mL	32.06	2.88	8.99
100 CFU/mL	38.67	1.28	3.32
1000 CFU/mL	51.38	2.40	4.66
10,000 CFU/mL	60.08	1.26	2.10

**Table 4:** Comparative analytical performance of recent biosensing platforms for bacterial detection.

Sensor Platform	Target	Recognition Element	Electrode	Linear Range	LOD	Reference
This work	*H. influenzae*	MIP	Gold SPE	1–10^4^ CFU/mL	1.03 CFU/mL	This study
Electrochemical aptasensor	*Salmonella* spp.	DNA aptamer	Carbon SPE	10–10^4^ CFU/mL	50 CFU/mL	[36]
MIP electrochemical sensor	*E. coli*	MIP	Glassy carbon	10–10^5^ CFU/mL	9.40 CFU/mL	[37]
Electrochemical immunosensor	*P. aeruginosa*	Antibody	Au electrode	10–10^6^ CFU/mL	8 CFU/mL	[38]

## Data Availability

The original contributions presented in this study are included in the article. Further inquiries can be directed to the corresponding author.
